# Safety of drainless excision of the submandibular gland^☆^

**DOI:** 10.1016/j.bjorl.2019.04.010

**Published:** 2019-05-21

**Authors:** Hae Sang Park, Sung Min Lee, Kang Hyun Lee, Mi Sun Chun, Han Su Kim

**Affiliations:** a Hallym University, College of Medicine, Chuncheon Sacred Heart Hospital, Department of Otorhinolaryngology-Head and Neck Surgery, Chuncheon, Republic of Korea; b Ewha Womans University, School of Medicine, Department of Otorhinolaryngology-Head and Neck Surgery, Seoul, Republic of Korea

**Keywords:** Fibrin glue, Drain, Submandibular gland excision, Cola de fibrina, Drenagem, Excisão da glândula submandibular

## Abstract

**Introduction:**

Percutaneous drains can be associated with several complications, including infection, fistula formation, discomfort and prolonged hospitalization.

**Objective:**

The aim of this study was to evaluate the safety of submandibular gland excision without the use of surgical drains.

**Methods:**

We analyzed the surgery time, postoperative complications such as bleeding, facial palsy, seroma, and repeat exploration of wounds and duration of the hospital stay. Excision of the submandibular gland via a transcervical approach was undertaken by two surgeons. Prior to wound closure, the skin flap and wound bed were approximated using hemostatic fibrin glue (Greenplast-Q PFS KIT®, GC Greencross, Youngin, Korea). Neither saline irrigation nor insertion of a percutaneous drain were included.

**Results:**

A total of 23 patients underwent submandibular gland excision. The study group consisted of 14 men (60.8%) and 9 women (39.2%) (mean age, 47.6 years; range, 24–70 years). There were two patients who had minor complications. One patient showed minor bleeding on the skin incision line immediately postoperatively, and one developed a seroma at 7 days postoperatively. There were no major surgical complications. Total duration of the surgery from skin incision to closure averaged 44.86 minutes. Mean duration of the hospital stay was 3.17 days. Patients were discharged on average at 1.17 days after surgery.

**Conclusion:**

The submandibular gland can be safely excised without the use of a surgical drain, therefore allowing early patient discharge.

## Resumo

**Introdução:**

Os drenos percutâneos apresentam várias complicações associadas, inclusive infecção, formação de fístulas, desconforto e permanência hospitalar prolongada.

**Objetivo:**

Avaliar a segurança da excisão da glândula submandibular sem o uso de drenos cirúrgicos.

**Método:**

Analisamos o tempo de cirurgia, as complicações pós-operatórias tais como sangramento, paralisia facial, seroma e necessidade de reexploração de ferida operatória e a duração da internação hospitalar. A excisão da glândula submandibular por via transcervical foi realizada por dois cirurgiões. Antes do fechamento da incisão, o retalho cutâneo e o leito da ferida operatória foram aproximados utilizando cola hemostática de fibrina (Greenplast-Q PFS KIT®, GC Greencross, Youngin, República da Coréia). Não houve irrigação salina nem uso de dreno percutâneo.

**Resultados:**

Foram submetidos 23 pacientes à excisão da glândula submandibular. O grupo de estudo consistiu em 14 homens (60,8%) e 9 mulheres (39,2%) (média de idade de 47,6 anos; variação de 24 a 70 anos). Dois pacientes apresentaram complicações menores. Um paciente apresentou pequeno sangramento na incisão da pele no pós-operatório imediato e um deles teve seroma aos 7 dias de pós-operatório. Não houve complicações cirúrgicas importantes. A duração total da cirurgia, desde a incisão na pele até o fechamento, foi de 44,86 minutos. A duração média da internação hospitalar foi de 3,17 dias. Os pacientes receberam alta em média 1,17 dia após a cirurgia.

**Conclusão:**

A glândula submandibular pode ser excisada com segurança sem o uso de dreno cirúrgico, permitindo que o paciente tenha alta hospitalar mais precocemente.

## Introduction

Excision of the submandibular gland (SMG) has been a well-established surgical procedure for treatment of neoplasms and non-neoplastic conditions, such as chronic sialadenitis, sialolithiasis, and ranula.^1^, ^2^ The traditional surgical procedure is usually performed through a 4–6 cm lateral transcervical skin incision.^1^, ^3^ However, this traditional approach sometimes results in an unsatisfactory scar. To avoid cosmetic problems, several additional surgical approaches to the SMG have been introduced: transoral, submental, and retroauricular.^1^, ^3^ These incisions are often assisted by endoscopy or a robotic instrument to complete the procedure.^1^, ^3^ Regardless of the surgical approaches, many surgeons usually insert percutaneous drains as postoperative management to avoid possible swelling caused by bleeding, which can lead to infection and is a potential risk to the airway.^4^ However, percutaneous drains have several associated complications, including infection, fistular formation, discomfort and a prolonged hospitalization.^5^, ^6^

Several studies have suggested that using hemostatic agents could replace the insertion of the surgical drain in head and neck surgeries, including thyroidectomy, and parotidectomy.^5^, ^7^, ^8^ However, there are few published studies on excision of the SMG, and only a few cases.^7^, ^8^ Fibrin glue, also referred to as fibrin sealant, is a surgical hemostatic agent derived from plasma coagulation proteins.^9^ Recently, it has been widely used in a number of surgeries, and has proved its safety. Fibrin glue achieves hemostasis by mirroring the final common pathway of the coagulation cascade. It can also be used for wound closure and tissue sealing.^5^, ^9^ Therefore, we hypothesized that the use of fibrin glue in the drainless SMG excision would be beneficial by reducing dead space through the tissue sealing effect as well as hemostasis.

In this study, we aimed to determine whether the drainless SMG excision combined with the use of a prophylactic hemostatic agent is a safe and feasible procedure by evaluating surgery time, postoperative complications (bleeding, facial nerve palsy, seroma, and repeat exploration of wounds) and duration of the hospital stay.

## Materials and methods

Twenty-three consecutive patients who had undergone SMG excision were retrospectively enrolled in this study at two tertiary training hospitals from March 2015 to March 2018. All procedures were undertaken by the two surgeons, who have more than 5 years of head and neck surgery experience. The surgical techniques and perioperative care did not differ. The study group consisted of 14 men (60.8%) and 9 women (39.2%) (mean age, 47.6 years; range, 24–70 years).

### Surgical protocol

A 4–6 cm standard incision was made along the lateral neck crease approximately 2 finger breadths below the lower margin of the mandible. After a subplatysmal skin flap was elevated, the facial vein and distal portion of the facial artery were identified and ligated at the inferior border of the SMG. They were reflected superiorly with the overlying fascia to preserve the marginal mandibular nerve. The mylohyoid muscle was retracted superiorly, then the lingual nerve, Wharton's duct, and hypoglossal nerves were identified. The submandibular ganglion, which was attached between the lingual nerve and the SMG, was divided. After the Wharton's duct was ligated, the proximal portion of the facial artery was identified and ligated. Following excision of the SMG, meticulous hemostasis was obtained. No saline irrigation was performed. Prior to wound closure, fibrin glue (Greenplast-Q PFS KIT®, GC Greencross, Youngin, Republic of Korea) was applied to the operative bed. The skin flap was then reapproximated to the operative bed and sustained pressure was applied for 30 s. The subcutaneous tissue void was closed using 4–0 Vicryl® (Johnson & Johnson, New Brunswick, NJ, USA). The skin was reapproximated using Steri-Strip® (3M, Maplewood, MN, USA) or Dermabond® (Johnson & Johnson, New Brunswick, NJ, USA). No drain was used, and a simple dressing was applied. All patients were planned to be discharged on the first postoperative day.

### Data collection

We analyzed the surgery time, postoperative complications (bleeding, facial palsy, seroma, and repeat exploration of wounds) and duration of the hospital stay. The volume of the excised SMG was obtained from the pathology report.

## Results

Table 1 shows the demographic and histologic characteristics of the 23 patients, among whom five had hypertension, and five had diabetes mellitus. One patient had been taking anticoagulation medication which was stopped seven days before surgery. Thirteen patients (56.5%) were diagnosed with pleomorphic adenoma and 7 (30.4%) with chronic sialadenitis. These 7 patients received SMG excision because of recurrent sialolithiasis or intraparenchymal stones.

**Table 1:** Demographic and histologic characteristics of the patients.

Category	*n* (%)
*Demographics*	
Male	14 (60.8)
Female	9 (39.2)
Total	23
Age (years) (mean ± standard deviation)	47.6 ± 11.5
Hypertension	5 (21.7)
Diabetes mellitus	5 (21.7)
Anticoagulation medication	1 (4.3)

*Histology*	
Pleomorphic adenoma	13 (56.5)
Chronic sialadenitis	7 (30.4)
Lymphoma	2 (8.8)
Schwannoma	1 (4.3)

Management of the facial artery and vein during the surgery are summarized in Table 2. All of the facial veins were divided using a Harmonic scalpel® (HS, Johnson & Johnson, New Brunswick, NJ, USA). Tie ligation was used in 11 patients (47.8%) for dividing the distal part of the facial artery (face) and a HS was used in 12 patients (52.2%). The proximal part of the facial artery (neck) was ligated in 21 patients (91.3%) and by a HS in 2 patients (8.7%). The range of the volume of the excised SMG was from 10.5 cm^3^ to 72.93 cm^3^ (Table 3).

**Table 2:** Type of ligation of facial artery and vein.

	Tie ligation, *n* (%)	Harmonic scalpel, *n* (%)
*Artery*		
Face	11 (47.8)	12 (52.2)
Neck	21 (91.3)	2 (8.7)

*Vein*		
Face	0	23
Neck	0	23

**Table 3:** Summary of the patients.

Patient number	Sex	Age	DM	Hypertension	Anticoagulation	Surgery time^a^	Complications^b^	Pathology	Size of the excised SMG (cm)	Duration of the hospital stay (day)	Duration of the hospital stay after surgery (day)
1	F	39	N	N	N	41	N	PA	4.3 × 3 × 2	3	1
2	M	36	N	N	N	39	N	PA	6.3 × 3.3 × 3	3	1
3	F	58	N	N	N	37	N	PA	4.5 × 4 × 1.8	3	1
4	M	24	N	N	N	38	N	PA	5.3 × 4.3 × 3.2	3	1
5	F	41	N	N	N	50	N	CSA	3.4 × 2.6 × 1.2	3	1
6	M	51	N	N	N	39	N	PA	4.5 × 3.5 × 2	3	1
7	M	47	N	N	N	53	N	CSA	4.5 × 3.5 × 2	4	2
8	F	39	N	N	N	35	Minor bleeding on skin incision line	CSA	3.5 × 2 × 1.5	4	2
9	F	58	N	Y	N	25	N	PA	2.9 × 2.3 × 1.9	4	2
10	M	39	N	N	N	39	N	PA	2.6 × 2	3	1
11	F	70	Y	Y	Y	46	N	CSA	4 × 3.5 × 1	3	1
12	M	50	N	N	N	35	N	PA	6 × 3.7 × 2.8	3	1
13	M	30	N	N	N	52	N	Lymphoma	5 × 4 × 2.6	3	1
14	M	53	Y	Y	N	45	N	CSA	4 × 3 × 2	3	1
15	F	54	N	N	N	42	N	Lymphoma	6 × 3 × 2.5	4	2
16	M	30	N	N	N	60	Seroma (POD 7, aspiration 3 times, 14cc, 34cc, 17cc)	Schwannoma	4 × 3 × 2	3	1
17	F	54	N	N	N	43	N	PA	4.7 × 2 × 2	3	1
18	M	60	N	N	N	35	N	PA	5 × 3.7 × 2.7	3	1
19	M	52	N	N	N	45	N	PA	5.5 × 3.3 × 3	3	1
20	F	57	Y	N	N	40	N	PA	3.2 × 2.5	3	1
21	M	54	N	N	N	60	N	CSA	4 × 3 × 2	3	1
22	M	59	Y	Y	N	70	N	CSA	4.2 × 3 × 1.5	3	1
23	M	40	Y	Y	N	63	N	PA	7 × 3.5 × 3.5	3	1

a Time from skin incision to closure.

b Postoperative bleeding, facial nerve palsy, seroma, repeat exploration. DM, diabetes mellitus; SMG, submandibular gland; F, female; M, male; N, no; Y, yes; POD, postoperative day; PA, pleomorphic adenoma; CSA, chronic sialadenitis.

After excising SMG, Surgicel® (Absorbable hemostat, oxidized regenerated cellulose, Johnson & Johnson, New Brunswick, NJ, USA) and Greenplast-Q PFS KIT® (hemostatic fibrin glue, GC Greencross, Youngin, Republic of Korea) were applied for hemostasis. Twenty-three patients (100%) used Greenplast-Q PFS KIT® and among these patients, eight patients also used Surgicel®.

A mean total surgery time from skin incision to closure was 44.86 ± 10.68 min. There were no major surgical complications, such as facial nerve palsy, postoperative bleeding, seroma, or repeat exploration of wounds. However, there were two patients who had minor complications. One showed minor bleeding on the skin incision line immediately postoperatively; it was easily controlled by bipolar cauterization. The other had a seroma at 7 days postoperatively. The seroma was aspirated 3 times at the OPD and resolved within 5 days. The amount of aspirated seroma was 14cc, 34cc and 17cc. The mean duration of the hospital stay was 3.17 days. The patients were discharged on average 1.17 days after surgery. The patients list is summarized in Table 3.

## Discussion

At present, fibrin glue has been used in several otolaryngology-head and neck surgeries, including facial plastic surgery,^10^ tonsillectomy,^11^ thyroidectomy,^8^ parotidectomy^5^, ^12^ and more complex head and neck surgeries (neck dissection, laryngectomy).^8^ Several researchers suggested that fibrin glue could substitute for drain insertion and decrease both the volume of postoperative wound drainage and the frequency of postoperative haematoma and seroma.^5^, ^12^

In this study, we investigated the safety and feasibility of the drainless SMG excision. There are only two published studies on excision of the SMG with no drain.^7^, ^8^ Among them, 17 cases of drainless SMG excision using Surgiflo® was reported by Bannister et al.^7^ However, they focused on the effectiveness of Surgiflo® and did not show the demographics and histologic characteristics of the patients, operation time, or duration of the hospital stay. Laverick et al. reported the amount of drainage after SMG excision to assess safety of the operation as day surgery.^4^ Their results show a clear trend in the pattern of drainage, which plateaued within 8 h postoperatively, with negligible drainage volume thereafter. In addition, 95% of the patients (57 among 60) drained 40 mL or less (mean 18 mL). In our study, we could confirm the volume of the excised SMG from the pathology reports. The volume of the excised SMG could represent the volume of dead space in the operation field. The range of the volume of the excised SMG was 10.5–72.93 cm^3^. Greenplast-Q PFS KIT®, which was used in this study, is the fast-setting fibrin glue composed of human fibrinogen concentrate, aprotinin, and thrombin. Prior to wound closure, we applied fibrin glue (Greenplast-Q PFS KIT®, GC Greencross, Youngin, Republic of Korea) into the operative bed. The skin flap was then reapproximated to the operative bed, and sustained pressure was applied for reducing dead space. We think that this process could reduce the volume of dead space, which reduced the volume of drainage from the wounds. In addition, fibrin glue itself can reduce the amount of drainage. Tisseel®, one of the fibrin glues, has been shown to reduce the volume of drainage from a wound in superficial and total parotidectomy.^12^

In this study, there were no major postoperative complications. However, there were two patients who had minor complications. Of these two patients, one showed minor bleeding on the skin incision line immediately postoperatively; it was easily controlled by bipolar cauterization. The other developed seroma at 7 days postoperatively. Seroma was aspirated 3 times at OPD and resolved within 5 days. Laverick et al. reported that 95% of the patients drained 40 mL or less after excision of the SMG. Additionally, they reported that 5% of the patients had a total drainage volume of more than 40 mL; these were currently taking an antiplatelet medication that had not been stopped or had undergone an emergency operation during an episode of acute infection.^4^ However, the two patients who had complications in this study showed neither anticoagulation medication nor acute infection. In addition, a patient who was admitted with acute sialadenitis caused by sialolithiasis one week prior to surgery was safely treated with the drainless SMG excision without any complications (Table 3, patient no. 21). We suggest that careful preoperative assessment, including consideration of anticoagulation medication and acute infection, is important for performing a safe drainless SMG excision. However, drainless SMG excision can be performed safely with meticulous hemostasis and prophylactic use of fibrin glue, even if acute infection is present.

In this study, the facial vein and artery were managed by various methods such as using HS or tie ligation. All of the facial veins and some cases of distal portion of the facial artery (face) (52.2%) were divided using a HS. However, most instances of proximal portion of the facial artery (neck) (91.3%) were divided and controlled by tie ligation. Although the safety of the HS has been demonstrated in other surgery such as thyroidectomy,^13^ we performed tie ligation for managing the proximal portion of the facial artery in order to prevent postoperative bleeding. However, in this study, the use of tie ligation or HS did not show any difference in postoperative bleeding. There was no postoperative bleeding regardless of the use of tie ligation or HS. In addition, in the case of patient who had postoperative seroma in this study, we used tie ligation for controlling proximal portion of the facial artery.

The mean duration of the hospital stay was 3.17 days in this study. All patients were planned to be discharged the first postoperative day. However, 3 patients (13%) were discharged on the second postoperative day at the patient's request. All patients were discharged on average 1.17 days after surgery and all the patients expressed satisfaction with their early discharge. In addition, patients who used Dermabond® for skin closure could take showers immediately after the surgery. From these results, we conclude that the drainless SMG excision could shorten hospital stays, thus reducing the financial burden, and increase patient satisfaction. Despite the fact that the drainless SMG excision with prophylactic fibrin glue showed promising results in this study, it has some limitations. First, because we designed this study as a retrospective study, it included a relatively heterogeneous group of patients and lacked a comparative control group. Second, relatively fewer cases were included in this study. Therefore, further prospective randomized, comparative studies with large numbers of patients are required.

## Conclusion

Drainless excision of the SMG can be safely performed with meticulous hemostasis and prophylactic fibrin glue, thus allowing patients to leave the hospital earlier.

## Ethical approval

This study was waived IRB approval; simple, non-invasive, retrospective medical record reviews.

## Conflicts of interest

The authors declare no conflicts of interest.

## References

[1] Beahm D.D., Peleaz L., Nuss D.W., Barry S., Jayc C.S., Carlos R.S. (2009). Surgical approaches to the submandibular gland: a review of literature. Int J Surg.

[2] Hernando M., Echarri R.M., Taha M., Martin-Fragueiro L., Hernando A., Plaza Mayor G. (2012). Surgical complications of submandibular gland excision. Acta Otorrinolaringol.

[3] Kim H.S., Chung S.M., Pae S.Y., Park H.S. (2011). Endoscope assisted submandibular sialadenectomy: the face-lift approach. Eur Arch Oto-Rhino-Laryngol.

[4] Laverick S., Chandramohan J., McLoughlin P.M. (2012). Excision of a submandibular gland: a safe day case procedure?. Br J Oral Maxillofac Surg.

[5] Al-Qahtani K. (2011). Initial experience with hemostatic fibrin glue as adjuvant during drainless parotidectomy. Saudi Dent J.

[6] Reid R.R., Dumanian G.A. (2003). A minimalist approach to the care of the indwelling closed suction drain: a prospective analysis of local wound complications. Ann Plast Surg.

[7] Bannister M., Ah-See K. (2014). Safety of the haemostatic agent Surgiflo® in excisions of the submandibular gland: our experience in 17 cases. Br J Oral Maxillofac Surg.

[8] Ujam A., Awad Z., Wong G., Tatla T., Farrell R. (2012). Safety trial of Floseal® haemostatic agent in head and neck surgery. Ann R Coll Surg Engl.

[9] Jackson M.R. (2001). Fibrin sealants in surgical practice: an overview. Am J Surg.

[10] Kenneth W.A., Shan R.B. (2003). Advances in facial rejuvenation surgery. Curr Opin Otolaryngol Head Neck Surg.

[11] Vaiman M., Eviatar E., Shlamkovich N., Segal S. (2003). Effect of modern fibrin glue on bleeding after tonsillectomy and adenoidectomy. Ann Otol Rhinol Laryngol.

[12] Maharaj M., Diamond C., Williams D., Seikaly H., Harris J. (2006). Tisseel to reduce postparotidectomy wound drainage: randomized, prospective, controlled trial. J Otolaryngol.

[13] Al-dhahiry J.K.S., Hameed H.M. (2016). Total thyroidectomy: conventional suture ligation technique versus sutureless techniques using harmonic scalpel or maxium. Ann Med Surg.

